# RNase III Participates in the Adaptation to Temperature Shock and Oxidative Stress in *Escherichia coli*

**DOI:** 10.3390/microorganisms10040699

**Published:** 2022-03-24

**Authors:** Maxence Lejars, Eliane Hajnsdorf

**Affiliations:** UMR8261, Institut de Biologie Physico-Chimique, CNRS, Université Paris Cité, 75005 Paris, France; maxence.lejars@md.tsukuba.ac.jp

**Keywords:** RNase III, heat shock, RpoH, oxidative stress, SodA

## Abstract

Bacteria thrive in ever-changing environments by quickly remodeling their transcriptome and proteome via complex regulatory circuits. Regulation occurs at multiple steps, from the transcription of genes to the post-translational modification of proteins, via both protein and RNA regulators. At the post-transcriptional level, the RNA fate is balanced through the binding of ribosomes, chaperones and ribonucleases. We aim to decipher the role of the double-stranded-RNA-specific endoribonuclease RNase III and to evaluate its biological importance in the adaptation to modifications of the environment. The inactivation of RNase III affects a large number of genes and leads to several phenotypical defects, such as reduced thermotolerance in *Escherichia coli*. In this study, we reveal that RNase III inactivation leads to an increased sensitivity to temperature shock and oxidative stress. We further show that RNase III is important for the induction of the heat shock sigma factor RpoH and for the expression of the superoxide dismutase SodA.

## 1. Introduction

In order to maintain fitness, bacteria sense environmental cues, and subsequently adapt their behavior by remodeling their transcriptome and proteome. Adaptation depends on regulatory mechanisms, which act at all levels of gene expression, from transcription to post-translation. At the post-transcriptional level, the fate of RNAs is decided by a plethora of effectors, e.g., small molecules, RNA-binding proteins and other RNAs. These regulators control the translation, protection and destabilization of messenger RNAs (mRNAs) via specific sequences and/or structural motifs (reviewed by [1,2,3]). Among these factors, ribonucleases (RNases) are major actors that ensure a tight control on gene expression (reviewed in [4]).

The endoribonuclease III (RNase III) domain, widely conserved in bacteria and eukaryotes, provides the specificity for double-stranded RNA (dsRNA) cleavage (reviewed in [5,6,7]). RNase III enzymes are involved in different processes, such as the repression and also activation of gene expression and maturation of stable and regulatory RNAs. However recent findings suggest that other functions could have emerged in some eukaryotic organisms, such as the direct involvement of DROSHA in the activation of transcription in humans [8]. In *Escherichia coli*, RNase III (encoded by the gene *rnc*) forms a dimer, and its canonical activity is to bind a dsRNA substrate that can either be intramolecular duplexes, formed within the same RNA molecule (e.g., hairpin loop) or intermolecular hybrids (e.g., antisense RNA bound to its complementary target) [7,9]. Upon binding to a specific target, the two RNase III monomers will adopt the active conformation and perform the cleavage of one or of both RNA strands, generating fragments whose ends are staggered by two bases on one strand compared to the other.

The role of RNase III in *E. coli* was first thought to be limited to the initial processing steps of rRNAs and to cleaving a few other specialized RNAs, such as SsrA and T7 mRNAs, to generate their mature and functional forms [10,11,12,13,14]. It later appeared that RNase III is more widely involved in the control of gene expression [15,16,17,18,19,20]. The dicistronic mRNA *rpsO-pnp*, encoding the ribosomal protein S15 and the exoribonuclease PNPase, was one of the first cases studied. RNase III cleavages in the 5′-untranslated region (UTR) of *pnp* initiate its degradation [16,18,21] and trigger further cleavages by RNase E, leading to the irreversible inactivation of the *pnp* mRNA [22]. As a consequence, inactivation of RNase III results in the overexpression of PNPase [23]. In a small number of cases, RNase III was also shown to be involved in the positive post-transcriptional regulation of a diverse set of genes, including *gadE* mRNA, encoding a regulator involved in acid resistance [24]; *adhE* mRNA encoding an alcohol dehydrogenase essential for fermentation [25]; *eno* mRNA encoding the metabolic enzyme enolase, which is also associated with the degradosome [26], *cIII* and *N* mRNAs from the phage λ [15,27] and the pre-mRNA of phage T7 [28]. Thus, in addition to its role in RNA decay, RNase III is also involved in the positive regulation of gene expression.

The application of RNA-seq methodologies has demonstrated an even wider role of RNase III in the control of gene expression [29,30,31]. One study, combining stability experiments with RNA-seq (i.e., sequencing after rifampicin treatment), reported 2054 RNase III-dependent cleavage sites (11 were verified on purified RNA fragments by in vitro RNase III cleavage assays) [32]. In another study, a tailored RNA-seq based approach coupled to an enrichment of small fragments in a mutant inactivated for RNase III (i.e., by the addition of a specific tag on 5′-monophosphate extremities after the degradation of 5′-triphosphate RNAs) detected 1003 cleavage sites in 615 targets [33]. Stead and colleagues observed, using a microarray-based experiment, that genes implicated in the response to heat constituted an important fraction of the total coding genes affected in the *rnc* mutant [30]. Another microarray study, performed in strains with reduced or increased RNase III levels, led to the identification of four genes negatively controlled by RNase III; *proP*, *proU*, *bdm* and *betT*, and revealed that RNase III activity is reduced during an osmotic shock and during a cold shock at the post-translational level (the latter caused by the binding of the YmdB protein to RNase III) [29,34,35,36,37]. A few other studies have addressed the roles of RNase III in *E. coli* physiology, revealing reduced thermotolerance and motility in an *rnc* mutant, while aminoglycoside-resistant mutant strains have been shown to rely on increased RNase III activity [38,39,40].

In this work, we show that the inactivation of RNase III is detrimental for survival at high and low temperatures and for resistance to oxidative stress. We observe that the induction of the heat shock sigma factor RpoH and the expression of the superoxide dismutase SodA are reduced upon inactivation of RNase III. We show that, during a heat shock, RNase III together with PNPase are required for the stabilization of the induced *rpoH* mRNA, while our results suggest that RNase III also plays a positive role in *rpoH* transcriptional induction. We further characterize the importance of RNase III and PNPase in the stabilization of *sodA* mRNA and highlight an additional role of RNase III in the transcriptional regulation of *sodA*.

## 2. Materials and Methods

### 2.1. Bacterial Strains, Growth Conditions

Strains, plasmids and primers used in this study are described in Table S1. RNase III, encoded by the *rnc* gene in *E. coli*, is co-expressed with the gene for the essential GTPase Era [41], and hence its deletion is not trivial, as demonstrated by the absence of an *rnc* mutant in the Keio collection [42]. We used the *rnc*105 mutation, a point mutation (G44D) leading to catalytic inactivation and reduced affinity for RNA [43] that has no polar effect on the transcription of the *era* gene, located downstream. Bacteria were grown in LB medium at 37 °C and sampled in mid-log phase (A_650_ ≈ 0.4) when not specified otherwise. The IBPC633 strain (carrying the *rnc*105 mutation linked to the *nadB51*::Tn10 (Tet^R^) mutation in the N3433 background) transformed with the pRNC1 plasmid expressing RNase III from the P*_ara_* promoter of the pKAN6 plasmid or the pKAN6 empty control vector [29] were grown in LB medium with 50 μg/mL kanamycin and induced by addition of arabinose at the indicated concentrations. N3433-P*_lac_*-*sodA* and IBPC633-P*_lac_*-*sodA* strains, transformed with the plasmid pBR*lacI^q^*, were grown in LB medium with 100 μg/mL ampicillin. Mutant alleles (*pnp*, *crp* and *cytR*) were moved to the N3433 and IBPC633 genetic background strains by P1 transduction.

### 2.2. Promoter Replacement and Strain Construction

The kmPcL genetic element, encoding the kanamycin resistance cassette followed by the P*_lac_* promoter, was amplified by PCR from the strain MG1655kmPcLyad with the primers mSodA3pClKan and Kan-pLac-SodA4 (Table S1) and inserted in front of the gene *sodA* at its native chromosomal location, as described in [44], with the following modifications. After purification, the cassette was electroporated into a strain containing an activated mini-λ expressing the λ-Red recombinase gene replacement system, allowing for the replacement by homologous recombination of the native promoter as described (Table S1) [45]. The modified P*_lac_*-*sodA* promoter was then moved by P1 transduction by selecting for resistance to kanamycin.

### 2.3. RNA Extraction and Northern Blot Analysis

Total RNA was prepared using the hot-phenol procedure [46]. Total RNA (5 µg) was electrophoresed on 1% agarose, 1xTBE gels for analysis by northern blot [22,47]. Membranes were hybridized with RNA probes synthesized by T7 RNA polymerase with [α-^32^P] UTP yielding uniformly labeled RNAs [48]. The size of RNAs was estimated by comparison with migration of the RiboRuler High Range marker (Thermo Scientific, Thermo Fisher Scientific, Waltham, MA, USA). RNA stability was measured on cultures treated with rifampicin (500 μg/mL). Total RNA was extracted after 40 s (time 0) and at the indicated time points. Membranes were also probed for M1 RNA (or 5S rRNA for oldest experiments) used as charge control.

### 2.4. Western Blot

Total protein samples were collected by centrifugation and pellets were resuspended in SDS-loading buffer containing DTT (Biolabs), sonicated and heat denatured. Total protein was separated after denaturation on a 4–15% mini Protean TGX gel, and the proteins were transferred on a nitrocellulose membrane using Trans-Blot system (Biorad, Hercules, CA, USA). Membranes were blocked for 1 h prior to overnight incubation with the anti-SodA (Invitrogen, Thermo Fisher Scientific, Waltham, MA, USA) or anti-RpoH (BioLegend, San Diego, CA, USA) antibodies that were diluted 1000-fold and 500-fold, respectively, in phosphate buffered saline (PBS) with 0.05% Tween 20. After washing and incubation with the secondary antibody, detection was performed using the Clarity Max reagent kit (Biorad, Hercules, CA, USA) and images acquired on a ChemiDoc (Biorad, Hercules, CA, USA). Protein sizes were estimated by comparison with migration of the PageRuler Plus 10–250 kDa ladder (Thermo Scientific, Thermo Fisher Scientific, Waltham, MA, USA). RpoH protein was identified as a single band on the membrane using monoclonal antibodies, whereas anti-SodA polyclonal antibodies revealed multiple bands. Therefore, the specific SodA protein was identified by comparison with a sample extracted from a *sodA* deleted strain (Figure S7C). Membranes were reprobed with anti-S1 antibodies (10,000-fold dilution) in order to use the ribosomal protein S1 as charge control.

### 2.5. In Vitro Processing by RNase III

A DNA template carrying a T7 promoter sequence was generated by PCR using primers mT7SodA and sodAterm (Table S1). The *sodA* template was transcribed using T7 RNA polymerase into the full-length *sodA* mRNA (772 nts), as described in [49]. RNA 5′-end labeling was performed with [γ-^32^P]-ATP using T4 polynucleotide kinase. Transcripts were incubated in 20 mM Tris acetate, pH 7.5, 10 mM magnesium acetate and 100 mM sodium acetate for 5 min at 37 °C and submitted to in vitro processing by RNase III of *E. coli* at 37 °C in the presence of 1 µg tRNA for 25 min with the indicated quantities of RNase III (Epicentre, Madison, WI, USA) [50]. After precipitation, addition of loading buffer and heat denaturation, samples were analyzed on 6% polyacrylamide (19/1)-urea 7 M-1xTBE gels along with 5′-radio-labeled *Msp*I digested pBR322 (New England Biolabs, Ipswich, Massachusetts, United States).

### 2.6. Data Analysis

Northern blots were scanned using a Typhoon FLA 9500 scanner (GE Healthcare, Chicago, IL, USA). The resulting .gel images were quantified using the ImageQuantTL software version 8.1. The acquired images were uniformly adjusted for their contrast before being cropped and assembled. Bands of interest were quantified and the abundance of the studied transcripts was normalized by comparison to the abundance of the M1 or 5S RNA. Northern blots were performed in technical duplicates or more, as indicated in legends. For the stability assay presented in Table 1, normalized abundance of the studied mRNAs at the indicated times after rifampicin treatments were plotted using linear regression with a 90% confidence level, half-life and standard deviations calculated using the Microsoft Excel software. Western blots were quantified as .tif images by measuring raw integrated density and background normalization using the ImageJ software version 1.53c (NIH, https://imagej.nih.gov/ij/, last accessed on the 22 March 2022) [51]. The abundance of the protein of interest was then normalized compared to the S1 loading control. The acquired images were uniformly adjusted for their contrast before being cropped and assembled. Survival assays and growth curves were performed in biological duplicates and a representative experiment is shown. Images of survival plates were captured in .tif format on a GelDoc (Biorad), uniformly adjusted for their contrast before being cropped and assembled using the ImageJ software version 1.53c.

## 3. Results

### 3.1. Survival under Extreme Temperatures Depends on RNase III

**Figure 1:**
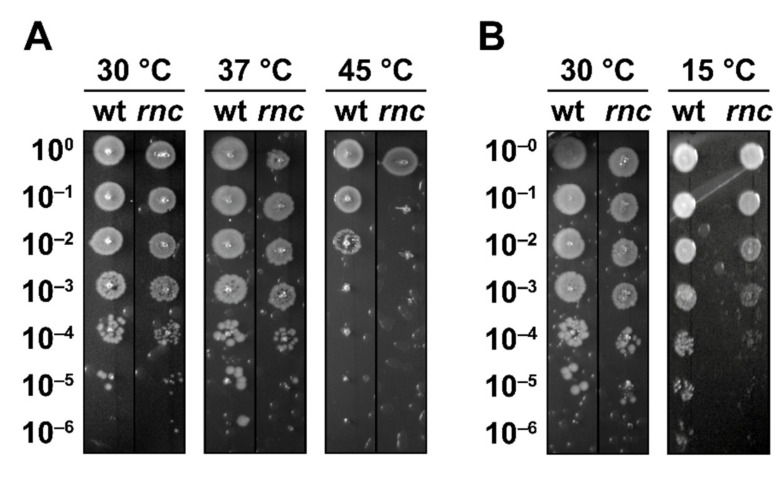
RNase III is required for survival at high and low temperatures. (**A**) N3433 (wt) and its *rnc*105 (*rnc*) derivative were grown at 30 °C before being shifted to 45 °C for 15 min, and survival was assayed by droplet plating at 30, 37 and 45 °C. (**B**) Strains wt and *rnc* were grown at 30 °C and survival was assayed by droplet plating at 30 and 15 °C.

We compared growth at different temperatures of a wild-type strain (N3433, referred to as wt) to its *rnc*105 derivative strain (IBPC633, referred to as *rnc*) by a droplet plating assay after a short heat shock (15 min at 45 °C). We observed comparable survival at 37 °C and at 30 °C, indicating that the number of viable cells is similar in the wt and *rnc* mutant (Figure 1A). In agreement with a previous study [38], the *rnc* mutant showed a decreased survival at 45 °C (Figure 1A). To demonstrate that RNase III inactivation is directly responsible for thermal sensitivity, we used the previously described pRNC1 plasmid, allowing for the ectopic expression of RNase III [29]. In the absence of induction, pRNC1 was shown to drive the expression of around 10% of the wt level and up to a 10-fold overexpression upon induction by arabinose [29]. In the *rnc* mutant, survival at 45 °C increases upon ectopic expression of RNase III from the pRNC1 plasmid, even in the absence of induction (Figure S1A), suggesting that a low level of wt RNase III is sufficient for phenotypic complementation.

Previous studies showed that RNase III is post-translationally inhibited upon the induction of YmdB during a cold shock, resulting in the induction of PNPase [37], whose expression is essential for survival during a cold shock [52]. Remarkably, we observed that RNase III inactivation led to a reduced survival at 15 °C (Figure 1B), a phenotype that has not been reported before. YmdB perturbs RNase III catalytic activity and homodimer formation both in vitro and in vivo, although the RNase III-YmdB complex still binds dsRNAs [53]. Thus, one possible explanation is that, while it is necessary to reduce the RNase III catalytic activity during cold shock, the RNase III-YmdB complex could still exert a regulatory role and coordinate the induction of a stress response. In agreement with this hypothesis, the *rnc*70 mutation was shown to impair the endonucleolytic activity of RNase III without affecting its ability to bind dsRNAs whereas *rnc*105 (used in this study) was suggested to have a reduced affinity for dsRNA [43].

In summary, these results demonstrate that RNase III is required by *E. coli* for sustained growth at high and at low temperatures.

### 3.2. RNase III Inactivation Increases Sensitivity to Hydrogen Peroxide

**Figure 2:**
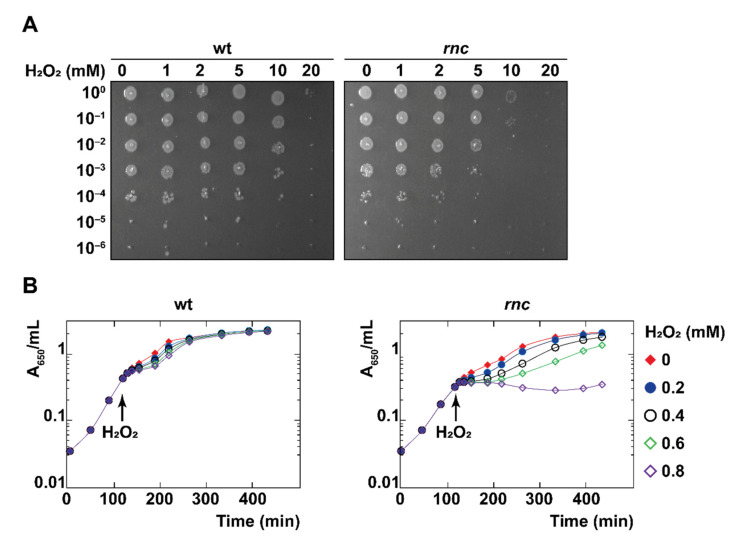
RNase III is required for survival under oxidative stress. (**A**) Strains wt and *rnc* were grown until mid-log phase, exposed to H_2_O_2_ at the indicated concentrations for 10 min, and survival was assayed by droplet plating. (**B**) Strains wt and *rnc* were grown at 37 °C and H_2_O_2_ was added to the cultures in mid-log phase, as indicated by a black arrow.

We challenged wt and *rnc* strains with hydrogen peroxide (H_2_O_2_), which is known to provoke a rapid, irreversible arrest of cell division due to the accumulation of DNA breaks [54]. A droplet plating assay after 10 min exposure to oxidative stress (from 5 mM H_2_O_2_) reveals a significant decrease in the survival of the *rnc* mutant (Figure 2A). Moreover, the ectopic expression of RNase III from pRNC1 increased the survival of the *rnc* mutant, even in the absence of induction, showing that even a low level of wt RNase III allows phenotypic complementation (Figure S1B), thus confirming that the expression of RNase III in the *rnc105* (*nadB51*::Tn10) mutant can restore resistance to oxidative stress. We confirmed that the effect of H_2_O_2_ (0.8 mM in liquid culture) on the survival of the *rnc* mutant was due to an immediate growth arrest (Figure 2B). We also challenged the two strains with paraquat (0.1 mM), which is known to generate superoxide [54], and observed a reduction in the survival of the *rnc* mutant after 60 min of exposure by a droplet plating assay (Figure S1C). However, paraquat challenge in liquid culture led to only a slight reduction in the growth rate of the *rnc* mutant (Figure S1D). Thus, RNase III inactivation leads to an increased sensitivity to oxidative stress mediated by H_2_O_2_ and, to a lower extent, mediated by paraquat.

### 3.3. The Induction of rpoH at High Temperature Depends on RNase III

**Figure 3:**
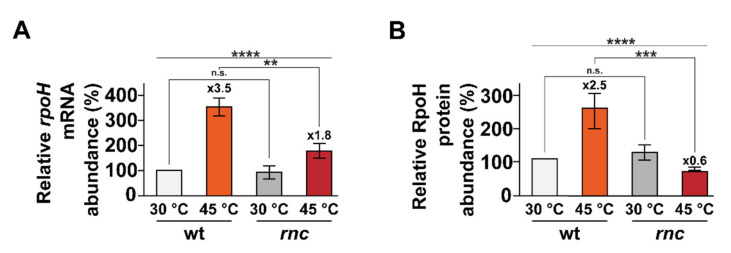
*rpoH* induction upon temperature upshift is defective in the *rnc* mutant. Strains wt and *rnc* were grown at 30 °C and transferred to 45 °C. Total RNA and protein sampled before or after a heat shock were analyzed by northern blot (see Figure S2) and Western blot (see Figure S3). (**A**) Mean *rpoH* mRNA levels and (**B**) mean RpoH protein levels are indicated before (30 °C, in grey) and after (**A**) 15 min or (**B**) 5 min of heat shock (45 °C, in red). Values are means of 3 biological replicates and error bars are standard deviations. Statistical significance was determined by ANOVA (n.s. for *p*-values ≥ 0.05, ** for *p*-values ≤ 0.01, *** for *p*-values ≤ 0.001 and **** for *p*-values ≤ 0.0001).

A previous transcriptomic approach revealed that a large number of genes belonging to the gene ontology group “response to heat”, including members of the heat shock RpoH regulon, are differentially expressed upon RNase III inactivation [30]. The heat shock sigma factor σ ^32^ (RpoH) is a key regulator of the *E. coli* heat shock response and is essential for growth at temperatures higher than 20 °C [55,56]. The expression of *rpoH* is induced upon a temperature upshift (among other conditions, see Discussion) via both enhanced synthesis and stabilization [57,58]. In particular the *rpoH* mRNA harbors an extensive secondary structure, located downstream from the start codon, containing four stem loops that act as a thermosensor controlling the translational efficiency in order to maintain a low translation of *rpoH* mRNA at temperatures less than 45 °C [58,59]. To examine whether RNase III has a role in the heat adaptation of *rpoH* expression, we measured *rpoH* mRNA and RpoH protein levels in the early (i.e., less than 15 min) response to heat shock [60,61,62]. Whereas the abundance of the *rpoH* mRNA increases after the heat shock (3.5-fold after 15 min of heat shock, Figure 3A and Figure S2), the amplitude of the induction was reduced twofold upon RNase III inactivation (1.8-fold after 15 min of heat shock). At the protein level, RpoH induction peaked after 5 min in the wt strain (2.5-fold after 5 min of heat shock, Figure 3B and Figure S3) whereas, unexpectedly, the abundance of RpoH decreased in the *rnc* mutant (0.6-fold after 5 min of heat shock). In brief, RNase III activity is critical for the induction of *rpoH* expression at both mRNA and protein levels during a heat shock, i.e., it has a positive role in controlling *rpoH* expression.

### 3.4. RNase III and PNPase Stabilize rpoH mRNA during a Heat Shock

As we observed a significant reduction in *rpoH* expression in the *rnc* mutant during a heat shock, we investigated the importance of RNase III on *rpoH* mRNA stability after a shift at 45 °C. We observed that RNase III inactivation led to a slight reduction in *rpoH* stability after a 15 min heat shock from 30 to 45 °C (from 4.01 to 3.39 min, Table 1 and Figure S4A), suggesting that RNase III has only a modest effect on the stability of the *rpoH* mRNA during a heat shock. However, it should be remembered that the inactivation of RNase III leads to the accumulation of the exoribonuclease PNPase, as previously shown at the mRNA [22] and protein level [23,31]. We observed that the stability of *rpoH* mRNA after a heat shock in a PNPase inactivated mutant (*pnp*) is reduced (from 4.01 to 3.14 min, Table 1 and Figure S4A). Hence, as PNPase is also involved in the stabilization of the *rpoH* mRNA after a heat shock, we reasoned that the elevated abundance of PNPase in the *rnc* mutant could mask the effect of RNase III. Supporting this hypothesis, we observed that the inactivation of RNase III in a *pnp* mutant led to a further reduction in *rpoH* mRNA stability after the heat shock (from 4.01 min to 2.06 min, Table 1 and Figure S4A). Thus, the stability of the *rpoH* mRNA after a heat shock relies on both RNase III and PNPase, and both ribonucleases have positive roles in the expression of *rpoH*.

### 3.5. RNase III Acts Independently from Transcription Factors CRP and CytR

As the above results show that the reduced *rpoH* induction during a heat shock is only partly due to the role of RNase III in the stabilization of *rpoH* mRNA (which is, moreover, partially compensated by the increase in PNPase), it appears that RNase III plays an additional positive role in the transcriptional regulation of *rpoH* during a heat shock, which is likely to be indirect via other effectors. The transcription of *rpoH* was shown to be dependent on the sigma factors RpoD, RpoE, RpoN and RpoS and regulated by transcriptional factors DnaA, ZraR, CpxR, IHF, CRP and CytR (Ecocyc database [41]). Remarkably, the details of the transcriptional induction of *rpoH* during a heat shock remains, to our knowledge, non-elucidated [31]. We compared the induction level of the *rpoH* mRNA after 15 min heat shock from 30 to 45 °C in mutants inactivated for CRP and/or CytR and/or RNase III. We observed that, while both *crp* and *cytR* mutations slightly affected the expression of *rpoH*, the reduction in *rpoH* induction in the RNase III mutant could still be seen in the absence of either or both *crp* and *cytR* (Figure S5). Hence, the role of RNase III in the induction of *rpoH* expression during a heat shock is independent of the transcription factors CRP and CytR.

### 3.6. Induction of Three Genes of the RpoH Regulon Is Defective in the rnc Mutant

**Figure 4:**
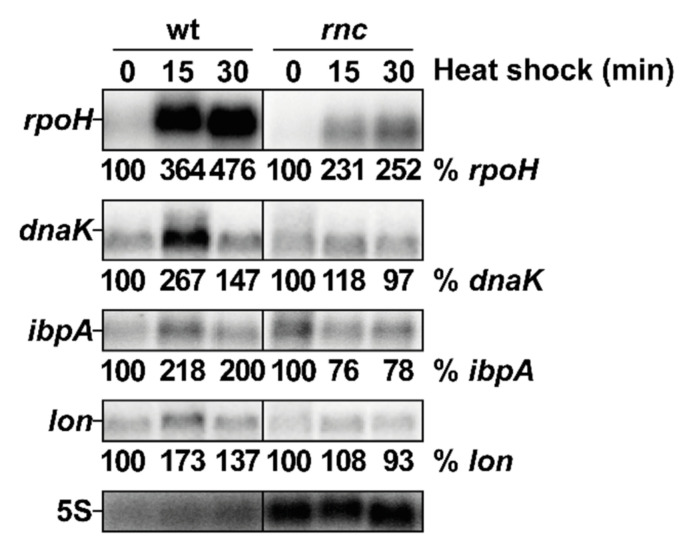
RNase III positively controls the induction of *rpoH* and of three targets of the RpoH regulon after heat shock. Strains wt and *rnc* were grown at 30 °C and shifted to 45 °C. Total RNA samples were taken before (time 0) or at the indicated time after the heat shock and analyzed by northern blot. The membrane was probed successively (after removal of previous signals) for *rpoH*, *dnaK*, *ibpA*, *lon* and 5S. Quantification of the different transcripts is given as % of the indicated mRNA in the wt at 30 °C normalized to the 5S rRNA charge control.

To investigate whether the inactivation of RNase III has repercussions on the RpoH regulon, we analyzed the effect of heat shock on three genes belonging to the RpoH regulon: *dnaK*, encoding a heat shock protein (HSP) chaperone, *ibpA,* encoding a small HSP chaperone and *lon*, encoding a major protease. In the wt strain, the expression of *dnaK*, *ibpA* and *lon* increased after 15 min at 45 °C and subsequently decreased after 30 min of heat shock, demonstrating that their induction is transient at the RNA level (Figure 4). In the *rnc* mutant, the *rpoH* mRNA abundance was lower (twofold) than in the wt, but was still induced by the heat shock, whereas the *dnaK*, *ibpA* and *lon* mRNAs levels decreased, which is in agreement with the previous observation that the RpoH protein does not accumulate during a heat shock in the *rnc* mutant (Figure 3B). This establishes a temporal correlation between RpoH protein levels and the expression of its regulon. Thus, RNase III is required for a full induction of *rpoH* and for at least three genes of its regulon (*dnaK*, *ibpA* and *lon*) during a heat shock.

### 3.7. RNase III Positively Regulates sodA Expression

**Figure 5:**
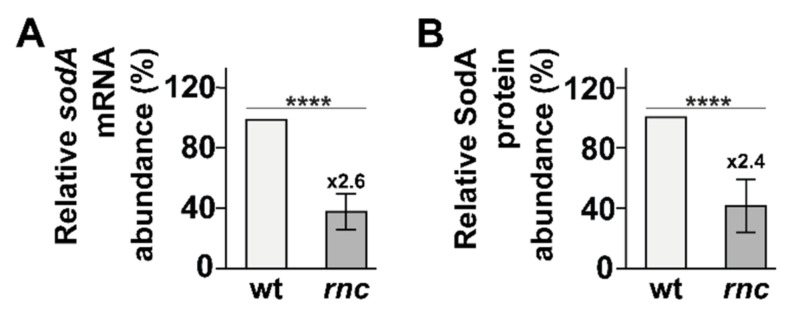
RNase III positively controls *sodA* expression. Total RNA and protein were sampled from strains wt and *rnc* and analyzed by (**A**) northern blot (see Figure S6) and (**B**) Western blot (see Figure S7). (**A**) Mean *sodA* mRNA levels and (**B**) mean SodA protein levels are shown. Values are means of 3 biological replicates and error bars are standard deviations. Statistical significance was determined by ANOVA (**** for *p*-values ≤ 0.0001).

RNase III is involved in the destabilization of the *sodB* mRNA, encoding the Fe^2+^-dependent superoxide dismutase B, upon binding by the sRNA RhyB under iron starvation [63]. More recently, the expression of *sodA*, encoding the major Mn^2+^-dependent cytoplasmic superoxide dismutase SodA, was observed to be strongly reduced in two independent *rnc* deletion mutants [31]. Thus, we investigated the role of RNase III in the regulation of *sodA* and in the resistance to oxidative stress. We first validated that *sodA* expression is reduced in the *rnc* mutant in the absence of stress at both mRNA (2.6-fold, Figure 5A and Figure S6) and protein levels (2.4-fold, Figure 5B and Figure S7). Furthermore, we observed that, after 10 min of oxidative stress in the presence of H_2_O_2_, *sodA* expression was slightly reduced at the mRNA level (Figure S6) but not at the protein level in the wt (Figure S7), while RNase III inactivation led to a strong reduction in *sodA* expression at both mRNA and protein level, similar to what could be observed in the absence of stress. Hence, RNase III is required for the expression of *sodA* in the absence of and during oxidative stress.

### 3.8. RNase III and PNPase Stabilize sodA mRNA

We then examined the stability of *sodA* mRNA and found that it was reduced in the *rnc* mutant (1.8-fold, Table 1 and Figure S4B). Since PNPase is upregulated in the *rnc* mutant strain, we examined whether the reduced stability of the *sodA* mRNA could be due to an increased expression of PNPase. The inactivation of PNPase destabilizes the *sodA* mRNA (2.3-fold, Table 1 and Figure S4B) and, together with the *rnc* mutation, leads to a further reduction (three-fold) in *sodA* mRNA stability. As RNase III has a role in the stabilization of *sodA* mRNA in the absence of stress, we investigated whether this effect could be due to an RNase III processing event that may stabilize the *sodA* mRNA. An in vitro RNase III cleavage assay showed that the full-length *sodA* mRNA is cleaved by RNase III at multiple positions, including one minor cleavage within the 5′-UTR (nts +28 relative to the transcription start site, Figure S8), which is similar to the unique in vivo cleavage site in the *sodA* mRNA previously identified [33]. In summary, RNase III is required for *sodA* mRNA stabilization and can cleave the *sodA* mRNA 5′-UTR in vitro, supporting previous in vivo observations. Hence, this suggests that, like in the case of the target mRNAs *gadE* [25] and *adhE* [25], RNase III can cleave the 5′-UTR of the *sodA* mRNA, which may lead to the stabilization of the transcript, either by protecting from other RNases or by improving the translation efficiency. The other cleavages observed in vitro, which were not detected in vivo, lie within the ORF and so might be protected from cleavage in vivo by translating ribosomes.

### 3.9. Transcriptional and Post-Transcriptional Regulation of the sodA Gene by RNase III

**Figure 6:**
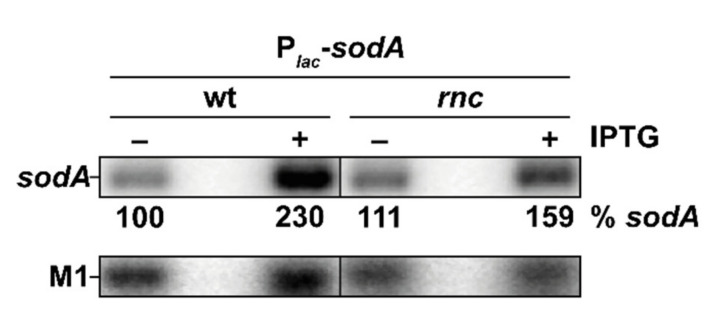
Transcriptional and post-transcriptional regulation of *sodA* by RNase III. N3433 P*_lac_*-*sodA* (wt) and IBPC633 P*_lac_*-*sodA* (*rnc*) strains with pBR*lacI^q^* expressing *lacI^q^* constitutively were induced (+) or not (−) with IPTG (0.1 mM) for 10 min. Total RNA was analyzed by northern blot; the membrane was probed for *sodA* and M1. Quantification of the *sodA* mRNA is given as % *sodA* mRNA in wt strain without IPTG.

We next looked for an effect of RNase III on the transcription as well as on the post-transcriptional regulation of *sodA*. To separate these regulatory layers, the endogenous *sodA* promoter was replaced by the *lacZ* promoter, which allows for an appreciable expression of *sodA* mRNA from the P*_lac_* promoter in the absence of IPTG (Figure 6). Contrary to the case where *sodA* is expressed from its own promoter, the *rnc* mutation had little effect on the *sodA* mRNA level. However, when strongly overproduced after IPTG induction, there was a slight reduction in the *sodA* mRNA level in the *rnc* mutant. This limited effect of RNase III on *sodA* mRNA expressed from an exogenous promoter, compared to the strong positive effect on *sodA* mRNA levels, when it is expressed from its own promoter, implies that the major role of RNase III in the regulation of *sodA* expression occurs by a transcriptional activation of the *sodA* gene. This indirect effect could be due to the regulation of an upstream regulator of *sodA* expression.

### 3.10. Overexpression of SodA in the rnc Mutant Supports Growth during Oxidative Stress

**Figure 7:**
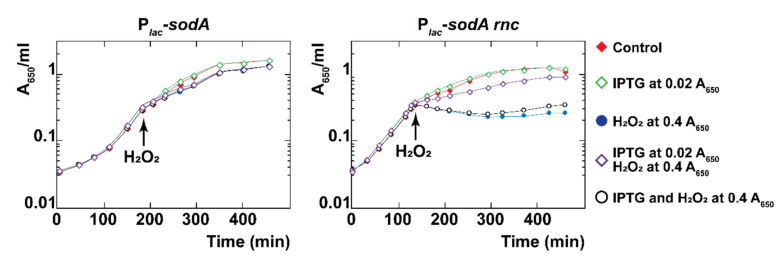
Ectopic expression of *sodA* protects from oxidative stress in the *rnc* mutant. N3433 P*_lac_*-*sodA* and IBPC633 P*_lac_-sodA* strains containing the pBR*lacI^q^* expressing *lacI^q^* constitutively were grown to mid-log phase, and IPTG (0.1 mM) and H_2_O_2_ (1 mM) were added as indicated. Red filled diamonds show the control with no additions; empty green diamonds show IPTG added at the beginning of the growth; blue filled circles show H_2_O_2_ added at mid-log phase; empty purple diamonds show IPTG added at the beginning of the growth and H_2_O_2_ at mid-log phase; empty black circles show IPTG added together with H_2_O_2_ at mid-log phase.

As in the case of *sodA* expressed from its chromosomal location (Figure 2B), there was little effect of the addition of H_2_O_2_ (1 mM) to the wt strain in mid-exponential growth expressing the basal level of SodA from P*_lac_*-*sodA* (Figure 7, left). In the *rnc* strain, H_2_O_2_ immediately stopped growth, and adding IPTG to induce SodA at the same time as the H_2_O_2_ challenge was not sufficient for maintaining growth. However, the constant overexpression of SodA from a low cell density was sufficient for maintaining growth after the H_2_O_2_ challenge in the *rnc* mutant (Figure 7, right). In summary, constant SodA overexpression can restore resistance to H_2_O_2_ in the *rnc* strain, suggesting that the RNase III-mediated positive regulation of *sodA* expression is required to protect against oxidative stress.

## 4. Discussion

**Figure 8:**
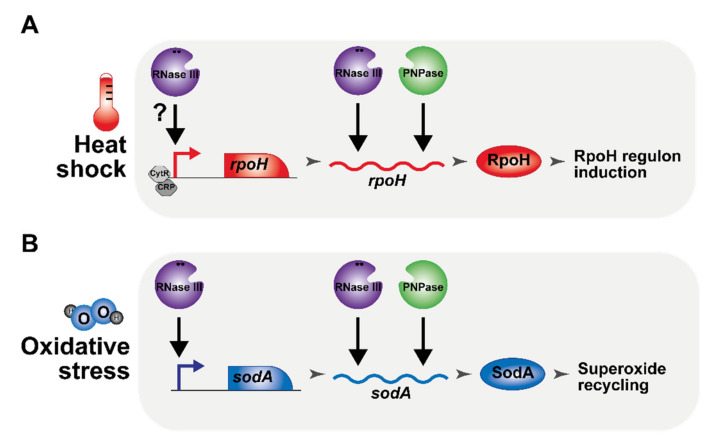
Schematic regulatory roles of RNase III in the adaptation to heat and to oxidative stress. (**A**) RNase III (purple) and PNPase (green) are required for the induction of *rpoH* (red) independently from the transcription factors CRP (dark grey) and CytR (grey). (**B**) RNase III and PNPase are important for the expression of the superoxide dismutase SodA (blue). Positive regulatory events (direct or indirect) are represented by a black arrow.

Recent global approaches, aimed at quantifying the importance of RNase III, have suggested that the range of RNase III targets had been considerably underestimated [29,30,31,32,33]. In this study, we demonstrate that the inactivation of RNase III increases sensitivity to heat and cold shock and to oxidative stress. We then show that the induction of *rpoH* during a heat shock is greatly impaired upon RNase III inactivation due, in part, to the requirement of RNase III, together with PNPase, in the stabilization of the *rpoH* mRNA. In addition, our results suggest that RNase III is required for the transcriptional activation of *rpoH* independently from the known transcriptional regulators of *rpoH* expression, CRP and CytR. Furthermore, we show that RNase III also positively regulates the expression of *sodA* at both the transcriptional and post-transcriptional levels. Significantly, we demonstrate that SodA overexpression restores growth to the *rnc* mutant under H_2_O_2_ stress. RpoH and SodA are, thus, new examples showing that RNase III is a positive regulator of gene expression. These new roles of RNase III are summarized in Figure 8.

### 4.1. Role of RNase III in Thermotolerance

*E. coli* adapts to heat shock through the transient induction of the RpoH regulon, leading to a short-term increase in HSPs in order to reduce heat shock damage (e.g., by expressing chaperones to counteract protein misfolding) [56]. Remarkably, it has been shown that RpoH and its regulon are induced under several other stress conditions, including carbohydrate starvation, hyperosmotic shock and growth in conditions of extracellular alkaline pH and in the presence of ethanol [56,64,65,66]. Furthermore, RpoH is required for long-term growth at 45 °C, and this thermotolerance remains poorly characterized. A proteomic analysis comparing *E. coli* strains grown in a bioreactor at 37 °C versus 45 °C revealed that among the most differentially expressed genes are the RpoH-dependent HSP DnaJ and the oxidative stress response factors SodA, AhpC and Dps [67]. Another study suggested that the early and transient peak of HSP expression plays an important role in the long-term remodeling of gene expression [68]. Other members of the RpoH regulon include ribosomal effectors, such as L31, and YbeY, an RNase involved in 16S rRNA maturation and quality control [69] that may suggest that thermotolerance also requires remodeling of the translation machinery. This hypothesis is corroborated by the fact that DnaJ, DnaK and other HSPs are instrumental in the maturation of ribosomes at 44 °C [70]. Thus, the reduced thermotolerance of the *rnc* mutant could result from the combined effects of the reduced *rpoH* induction and also from an alteration of rRNA maturation.

### 4.2. Importance of RNase III in the Positive Regulation of Gene Expression

RNase III is involved in mRNA destabilization, as in the case of the *rpsO*-*pnp* operon, where a dsRNA cleavage in the intergenic region enables the degradation of the *pnp* mRNA via RNase E [22]. Examples of destabilization by RNase III include, but are not limited to, the *rnc*-*era* operon [16], *proU* [35], *betT* [34], *bdm* [29], *proP* [36] and *rng* [39] mRNAs. However, RNase III was also shown to play a role in mRNA stabilization, as in the case of the mRNA *adhE* [25], where a dsRNA cleavage permits efficient translation and increases the mRNA stability. During acid stress, the asRNA ArrS is induced and binds to the *gadE* mRNA [24], triggering a maturation event by RNase III, leading to an increased translation and stability. In the case of the precursor mRNA of the T7 phage [28,71], RNase III was shown to proceed to single strand cleavages, releasing individual mRNAs with a stable hairpin in their 3′-ends. Other cases of positive regulation by RNase III have been identified, but the mechanism has not been elucidated, such as *eno* [26], *ahpC*, *pflB*, *yajQ* [32] and *sucA* [72] mRNAs. The importance of RNase III in the positive regulation of gene expression is further suggested by two independent transcriptomic analyses showing that 23% (120 out of 511 [30]) and 47% (87 out 187 [29]) of the genes whose expression was altered in an *rnc* mutant were downregulated. Furthermore, the identification of RNase III cleavage sites in vivo revealed that the RNase III targetome is even larger than previously suspected (615 targeted RNAs, including the 5′-UTR of *sodA* mRNA) [33]. In this work, we show that *rpoH* (during a heat shock) and *sodA* are new examples demonstrating a role of RNase III in the positive regulation of gene expression. The identification of new targets positively regulated by RNase III is limited by two aspects: first, RNase III inactivation leads to a large increase in PNPase expression, which could mask the direct role of RNase III in gene expression. Second, since our results and previous studies show that RNase III is implicated in various stress responses (heat and cold shock, oxidative stress, osmotic choc [29] and antibiotic resistance [39]), further studies aimed at identifying targets of RNase III would benefit from transcriptomic and proteomic analyses performed under stress conditions comparing strains inactivated for both RNase III and PNPase.

### 4.3. RNase III Is a General Stress Response Regulator and a Potential Target to Control Bacterial Virulence

In *E. coli*, the post-translational inhibition of RNase III by the protein YmdB is required for the resistance to osmotic choc and leads to an increase in biofilm formation through the stabilization of *bdm*, *proV*, *proW*, *proX*, *proP* and *betT* mRNAs [29,34,35,36]. On the contrary, RNase III activity is required for resistance to low concentrations of aminoglycosides via the repression of RNase G expression [39]. In addition, we show that RNase III is required for survival under heat and cold shock and for oxidative resistance. Remarkably, in *Listeria monocytogenes*, RNase III was similarly shown to be critical for resistance to heat and cold shock, to high and low pH, to oxidative stress and for survival in macrophages [73]. Hence, RNase III is an important factor in the cell’s ability to coordinate the cellular response toward different stress conditions.

The general stress response has been defined as the activation of a resistance response to multiple stresses after the sensing of a single stress, for which, the sigma factor RpoS is the primary regulator [73]. It is noteworthy that previous studies have reported a role for RNase III in the regulation of RpoS. Nevertheless, there were discrepancies in the outcome of this regulation in the different studies, which may result from differences in the experimental conditions and/or multiple regulatory mechanisms involving RNase III [31,74,75,76]. However, as in the case of RpoS [74], the absence of RNase III under standard conditions already affects the expression of a wide range of catabolic genes, as well as stress functions. RNase III can thus be considered as another general stress response regulator [75] and an important component of the complex mechanisms determining the ability of *E. coli* to adapt to ever-changing environmental conditions. Furthermore, the function of RNase III as a general stress response factor is likely to be conserved in other organisms, as demonstrated in the case of *L. monocytogenes* [73].

RNase III is a well-conserved protein among bacteria and was shown to be important for virulence in a wide range of pathogenic organisms, such as *Staphylococcus aureus* in the infection of mice [77], *Salmonella enterica* serovar Typhimurium [78] and *Enterococcus fecalis* [79] in the infection of *Galleria mellonella*. Hence, RNase III could represent an interesting target for therapeutic purposes. However, we want to stress that two contra-indicatory points should be considered: first RNase III inhibition in *E. coli* increases the sensitivity to temperature and oxidative stress but also increases resistance to osmotic choc and favors biofilm formation, which may lead to adverse outcomes that could preclude clinical uses. Second, targeting RNase III will be difficult to limit to a single species and will affect a whole range of species due to the strong conservation of RNase III in bacteria (reviewed in [5]). Thus, the use of RNase III modulators would have to be carefully assessed in a clinical setting.

## 5. Conclusions

We have extended our understanding of *E. coli* stress responses by showing that RNase III is required for the expression of RpoH after a heat shock and for the expression of SodA, thus allowing bacteria to cope with oxidative stress. Hence, RNase III is an important factor in the cell’s ability to coordinate the cellular response toward different stress conditions.

## Figures and Tables

**Table 1:** Effect of *rnc* and *pnp* mutations on the decay rates of *rpoH* and *sodA* mRNAs.

Transcripts	Temperature	wt	*rnc*	*pnp*	*rnc pnp*
*rpoH*	45 °C	4.01 ± 0.04	3.39 ± 0.08	3.14 ± 0.05	2.06 ± 0.09
*sodA*	37 °C	4.72 ± 0.09	2.57 ± 0.05	2.02 ± 0.10	1.50 ± 0.13

N3433 (wt), N3433-*pnp* (*pnp*) and their *rnc*105 (*rnc* and *rnc pnp*) derivatives were grown at 30 °C and transferred at 45 °C for 15 min (*rpoH*) or grown at 37 °C (*sodA*) before addition of rifampicin. Total RNA was sampled at the indicated times after rifampicin addition and analyzed by northern blot (Figure S3). Half-lives (minutes) were calculated as described in the material and methods.

## Supplementary Materials

The following are available online at https://www.mdpi.com/article/10.3390/microorganisms10040699/s1, Table S1: Strains, plasmids and primers, Figure S1: RNase III is required for heat shock and oxidative stress resistance, Figure S2: *rpoH* induction upon temperature upshift is defective in the *rnc* mutant at the mRNA level, Figure S3: *rpoH* induction upon temperature upshift is defective in the *rnc* mutant at the protein level, Figure S4: Effect of *rnc* and *pnp* mutations on the decay rates of *rpoH* and *sodA* mRNAs, Figure S5: RNase III inactivation reduces *rpoH* induction after heat shock independently from CRP and CytR, Figure S6: RNase III positively controls *sodA* expression at the mRNA level, Figure S7: RNase III positively controls *sodA* expression at the protein level, Figure S8: In vitro cleavage of *sodA* mRNA by RNase III and uncropped gels from Figure 4, Figure 6, Figures S3, S5 and S7. References References [80,81,82,83,84] are cited in the Supplementary Materials.
