# Supplementary material for: RNase III Participates in the Adaptation to Temperature Shock and Oxidative Stress in Escherichia coli
Source: Microorganisms. 2022 Mar 24;10(4):699. doi: 10.3390/microorganisms10040699 (PMC9032294; doi:10.3390/microorganisms10040699)
Supplement: Supplementary file 1 [file microorganisms-10-00699-s001.zip › microorganisms-1620923-supplementary.pdf]

microorganisms

MDPI

Supplementary Materials

RNase III Participates in the Adaptation to Temperature Shock and Oxidative Stress in *Escherichia coli*

Maxence Lejars † and Eliane Hajnsdorf *

UMR8261, Institut de Biologie Physico-Chimique, CNRS, Université Paris Cité, 75005 Paris, France; maxence.lejars@md.tsukuba.ac.jp
* Correspondence: eliane.hajnsdorf@ibpc.fr; Tel.: +33-158415126
† Current address: Transborder Medical Research Center, Faculty of Medicine, University of Tsukuba, Tsukuba 3058575, Japan

1. Supplementary Table

Table S1. Strains, plasmids and primers.

Strain	Relevant Characteristics	Source or Reference
N3433	HfrH *lacZ43 relA1 spoT1 thi1*	[80]
IBPC633	N3433 *rnc105 nadB51::*Tn10 (Tet^R)*	[81]
CA244-*pnp*Tn5	*pnp::*Tn5(Km^R)	[82]
MG1280	MG1655 *crp::cat* (Cm^R)	M. Guillier
JW3905	*ΔcytR721::kan* (Km^R)	Keio collection
JW3879	*ΔsodA768::kan* (Km^R)	Keio collection
N3433-*pnp*	N3433 *pnp::*Tn5 (Km^R)	This work
IBPC633-*pnp*	IBPC633 *pnp::*Tn5 (Km^R, Tet^R)	This work
N3433-*crp*	N3433 *crp::cat* (Cm^R)	This work
N3433-*cytR*	N3433 *ΔcytR721::kan* (Km^R)	This work
N3433-*crp-cytR*	N3433 *crp::cat ΔcytR721::kan* (Cm^R, Km^R)	This work
IBPC633-*crp*	IBPC633 *crp::cat* (Cm^R)	This work
IBPC633-*cytR*	IBPC633 *ΔcytR721::kan* (Km^R)	This work
IBPC633-*crp-cytR*	IBPC633 *crp::cat ΔcytR721::kan* (Cm^R, Km^R)	This work
MG1655kmPcLyad	yadNecpDhtrEyadMLKC under the control of the kmPcLrbs cassette λP_R promoter (Km^R)	[44]
ML69	MG1655-B mini λ, (Tet^R)	This work
N3433-P_{lac}-*sodA*	N3433 P_{lac}-*sodA*, pBR*lacIq* (Km^R, Amp^R)	This work
IBPC633-P_{lac}-*sodA*	IBPC633 P_{lac}-*sodA* (Km^R), pBR*lacIq*, *rnc105* (Km^R, Tet^R, Amp^R)	This work

Plasmid	Relevant characteristics	Source or Reference
pBR*lacI^q*	pBR322 with Constitutive *lacIq*, AmpR	M. Guillier

pRNC1	pKAN6 plasmid containing *rnc* under an arabinose-inducible promoter (Km^R)	[29]
pKAN6	Control vector	[29]

**Primers**	**Sequence**
Strain construction	
mSodA3pClKan	CCTGCAAAACCATACCCTTACGAAAAGTACGGCATTGATAATCATTCGCTCAAGTTAGTAATTCTCAC
Kan-pLac-SodA4	GTCGGGCGGCCGATTGTTAATGCCGCGTAAGCAGTGTGCTCAGTATCTTGTTATCCGCTCACAATGTCAATGTTGATA TCCGCTCACATTTATTAGTACATGCAACCATTATCAC
Northern blot probing	
mRpoH1	GCGCCAGAAGATATCGATTG
T7RpoH2	TAATACGACTCACTATAGGGTCGCAACTTTGACGATAC
mDnaK1	AACCGCAGTGAGTGAGTCT
T7DnaK2	TAATACGACTCACTATAGGGCGTCTCCACTATATATTCGG
mSodA1	ATGAGCTATACCCTGCCATC
T7SodA2	TAATACGACTCACTATAGGGTTAGCGTGACCGCCAGC
mIbpA1	ATGCGTAACTTTGATTTATCCC
T7IbpA2	TAATACGACTCACTATAGGGTGCGCTCTTTTTGTTCGTC
mLon1	CCTGAGCGTTCTGAACGCATTG
T7Lon2	TAATACGACTCACTATAGGGTGCCGTCAGGCAGTTTCAG
M1	GCTCTCTGTTGCACTGGTCG
5S	ACTACCATCGGCGCTACGGC
In vitro processing	
mT7SodA	TAATACGACTCACTATAGGGACTGCTTACGCGGCATTAACAATCGG
sodAterm	TTATTTTGAAATTGATCACAAAAAAACCACCGCCGTTGGCGATGGTTC

* It has been brought to our attention that the *nadB* gene, encoding L-aspartate oxidase has been implicated in the formation of endogenous H_2O_2 in *E. coli* by Korshunov and Imlay [83]. We wish to stress that as the cloned *rnc* gene fully complements the effects associated with the *rnc* mutation in our tests of survival to oxidative stress, any contribution of the *nadB* mutation to the phenotypes we observe is minimal.

2. Supplementary Figures

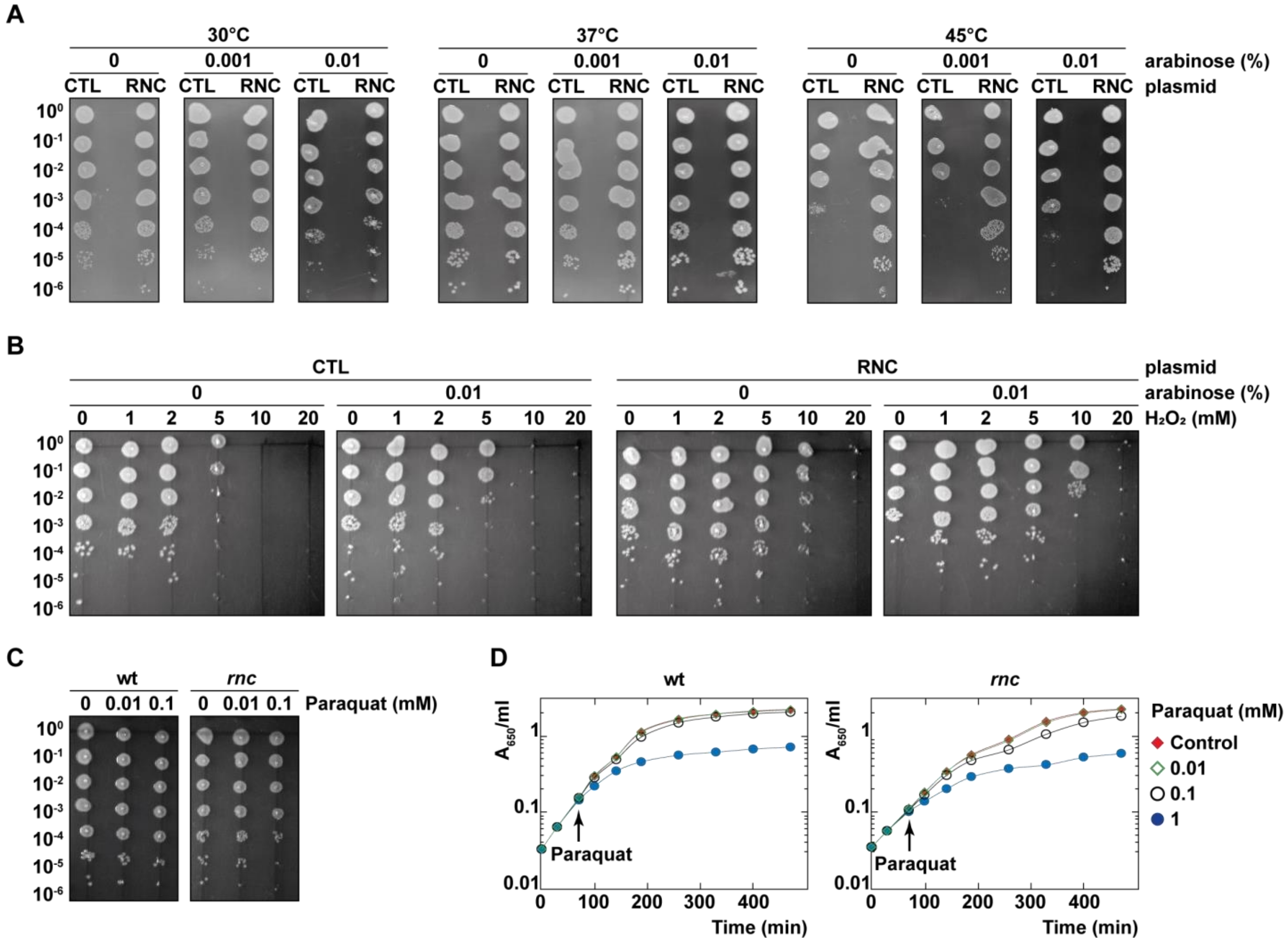

Figure S1. RNase III is required for heat shock and oxidative stress resistance. (**A**,**B**) IBPC633 (*rnc*) was transformed with the pKAN6 control plasmid (CTL) or pRNC1 (pKAN6 containing a cloned copy of *rnc* under an arabinose-inducible promoter (RNC). (**A**) Serial dilutions were spotted on LB plates containing kanamycin and the indicated concentrations of arabinose. Plates were incubated at 30 °C, 37 °C and 45 °C. (**B**) Bacteria were treated, or not, with H_2O_2 as indicated for 10 min, then serial dilutions were spotted on LB plates containing kanamycin and arabinose as indicated. (**C**) Strain N3433 (wt) and its *rnc*105 derivative (*rnc*) were treated with paraquat as indicated. Bacterial growth was continued for 60 min after paraquat addition and samples were diluted and spotted on LB plates. Plates were incubated at 37 °C. (**D**) Strains wt and *rnc* were grown to mid-log phase and paraquat was added as indicated by a black arrow.

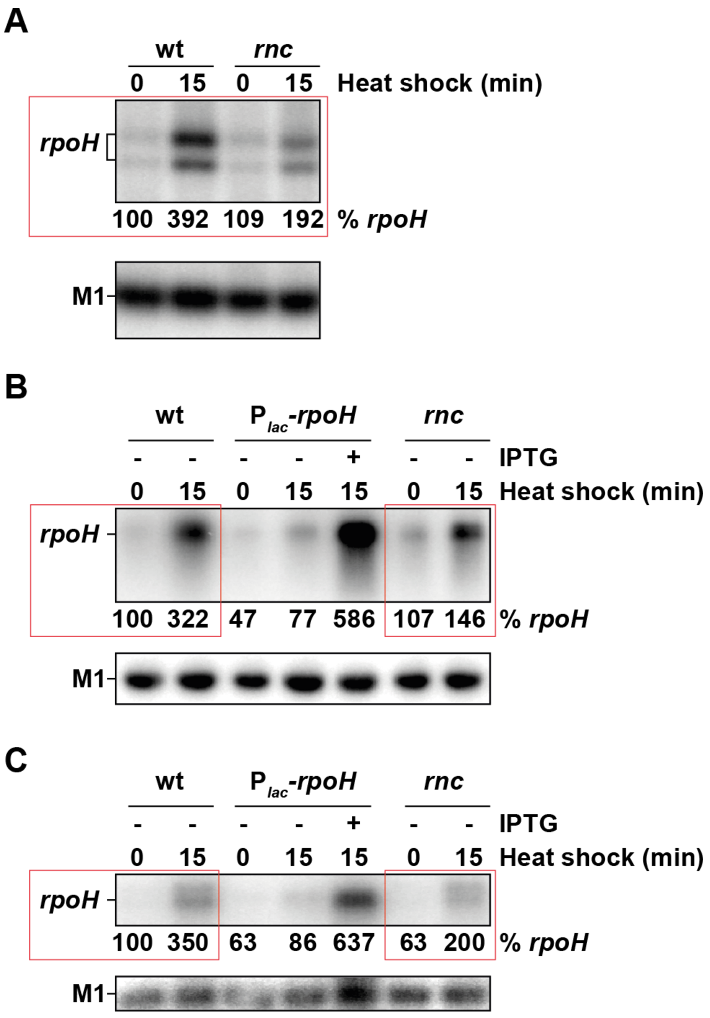

Figure S2. *rpoH* induction upon temperature upshift is defective in the *rnc* mutant at the mRNA level. Strains wt and *rnc* were grown at 30 °C and transferred to 45 °C. Total RNA sampled before or after the heat shock, at the indicated times, was analyzed by northern blot. Membranes were probed successively for *rpoH* and M1. Quantification is given as % of the wt at 30 °C. Quantified lanes used in Figure 2—(**A**) are boxed in red. (**B**,**C**) The P_{lac}-*rpoH* corresponds to a replacement of one of the endogenous P_{rpoH} by a P_{lac} promoter but does not completely abrogate *rpoH* transcriptional regulation hence is not discussed in the main text.

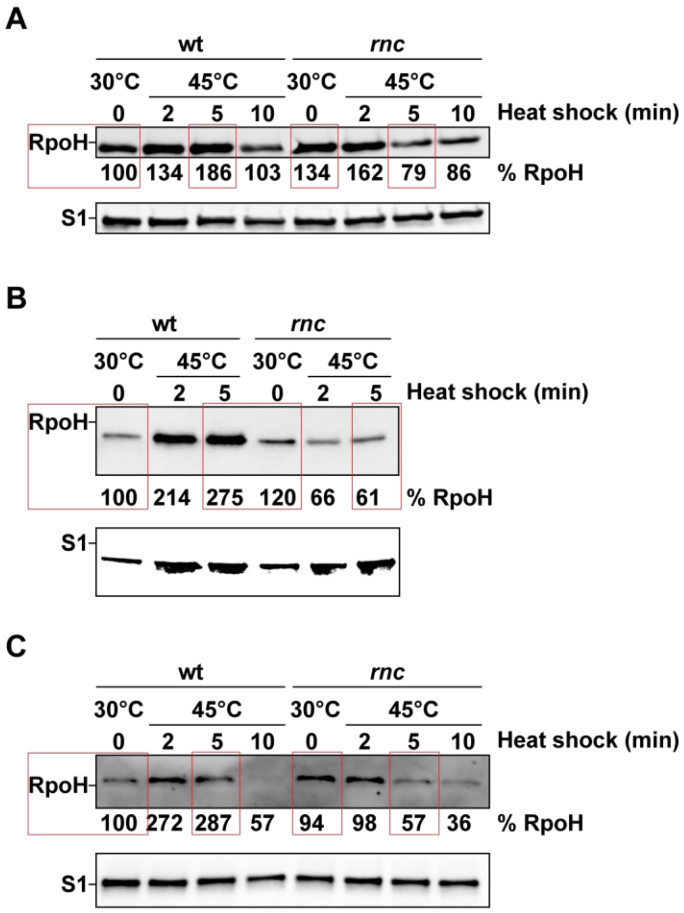

Figure S3. *rpoH* induction upon temperature upshift is defective in the *rnc* mutant at the protein level. Strains wt and *rnc* were grown at 30 °C and transferred to 45 °C. Total protein sampled before or after the heat shock, at the indicated times was analyzed by western blot. Membranes were probed successively for RpoH and S1. Quantification is given as % of the wt at 30 °C. Quantified lanes shown in Figure 2-B are boxed in red.

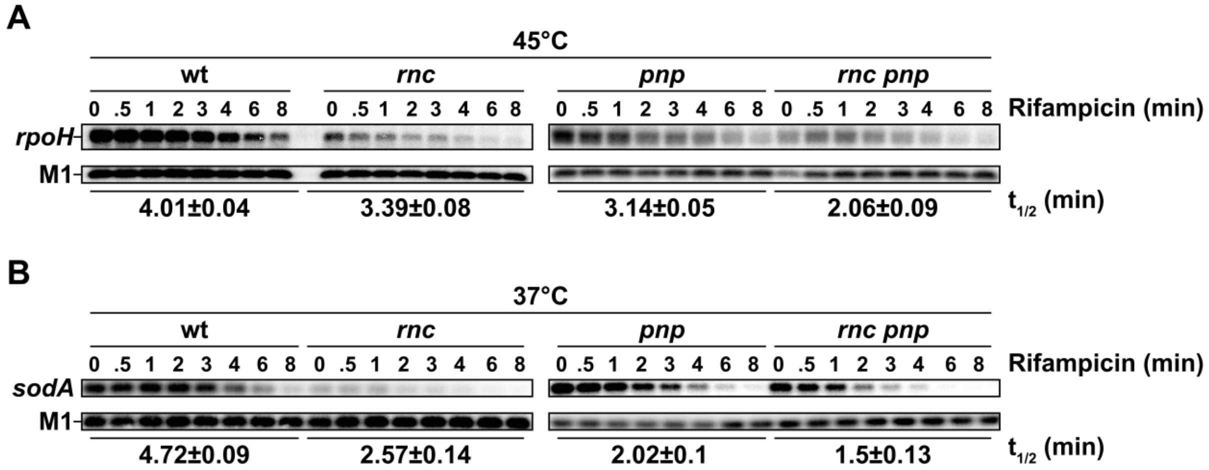

Figure S4. Effect of *rnc* and *pnp* mutations on the decay-rates of *rpoH* and *sodA* mRNAs. N3433 (wt), N3433-*pnp* (*pnp*) and their *rnc*105 (*rnc* and *rnc pnp*) derivatives were grown at (**A**) 30 °C and transferred to 45 °C for 15 min or (**B**) at 37 °C. Total RNA was sampled at the indicated times after rifampicin addition and analyzed by northern blot. Membranes were probed successively fo r (**A**) *rpoH* and M1 and (**B**) *sodA* and M1. Half-lives ($t_{1/2}$) were calculated as described in the material and method after quantification of the *rpoH* or *sodA* mRNAs normalized relative to M1 RNA.

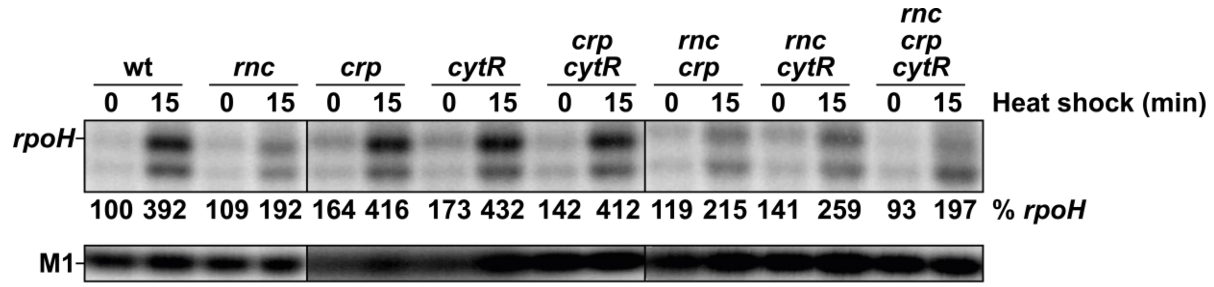

Figure S5. RNase III inactivation reduces *rpoH* induction after heat shock independently from CRP and CytR. N3433 (wt), N3433-*crp* (*crp*), N3433-*cytR* (*cytR*), N3433-*crp-cytR* (*crp cytR*) and their *rnc*105 (*rnc*, *rnc crp*, *rnc cytR* and *rnc crp cytR*) derivatives were grown at 30°C and transferred to 45 °C. Total RNA was sampled before or 15 min after the heat shock and analyzed by northern blot. The membrane was probed for *rpoH* and M1. Quantification of the *rpoH* mRNA (normalized relative to M1) is given as % *rpoH* mRNA in wt at 30 °C.

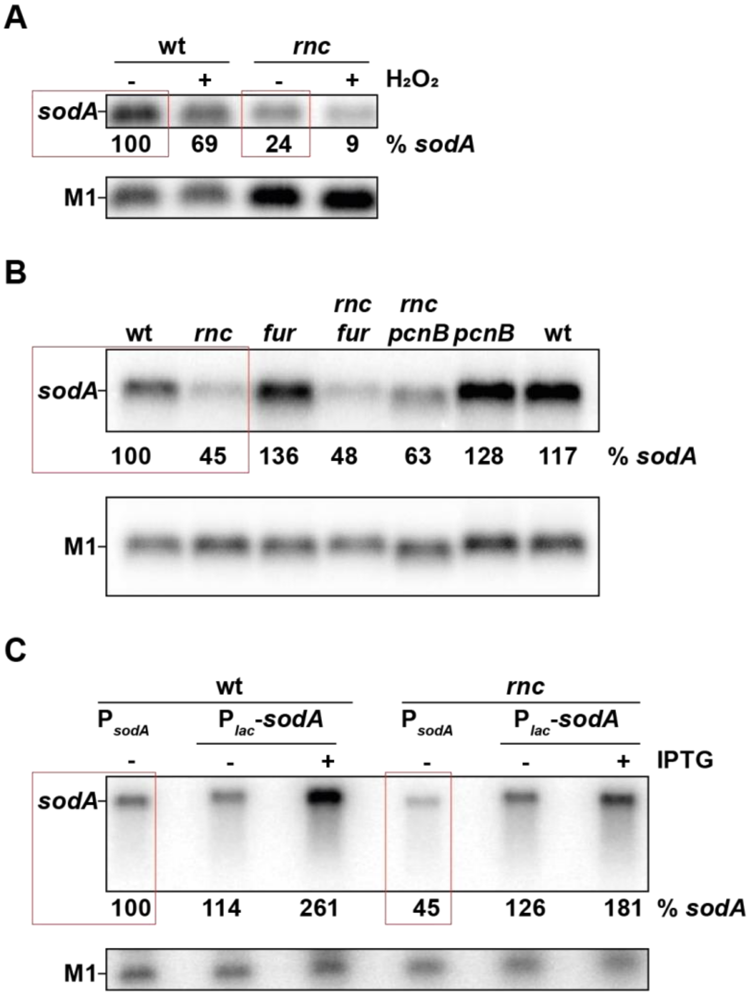

Figure S6. RNase III positively controls *sodA* expression at the mRNA level. Total RNA was sampled from strains wt and *rnc* and analyzed by northern blot. Membranes were probed successively for *sodA* and M1. Quantification is given as % of the wt in the absence of stress. Quantified lanes shown in Figure 5-A are boxed in red.

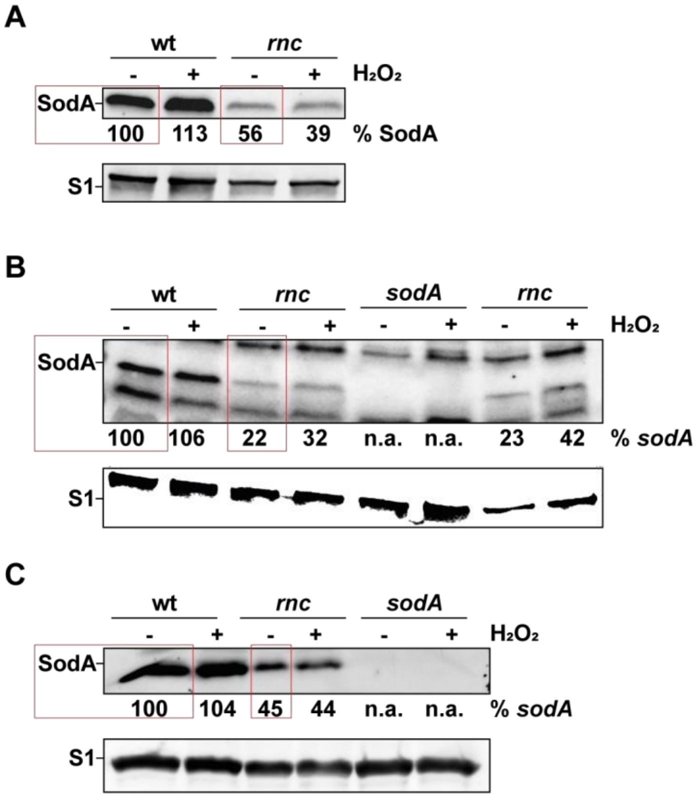

Figure S7. RNase III positively controls *sodA* expression at the protein level. Strains wt and *rnc* were grown until mid-log phase and transferred to new flasks containing (+) or not (-) H_2O_2 (10 mM) and sampled after 10 min. Total protein was analyzed by western blot. Membranes were probed successively for SodA and S1. Quantification is given as % of the wt in the absence of stress. Quantified lanes shown in Fig. 5-B are boxed in red. B and C) A *sodA* deletion mutant (*sodA*) from the Keio collection (JW-3879) was analyzed to control for the specificity of the SodA antibody as mentioned in the text.

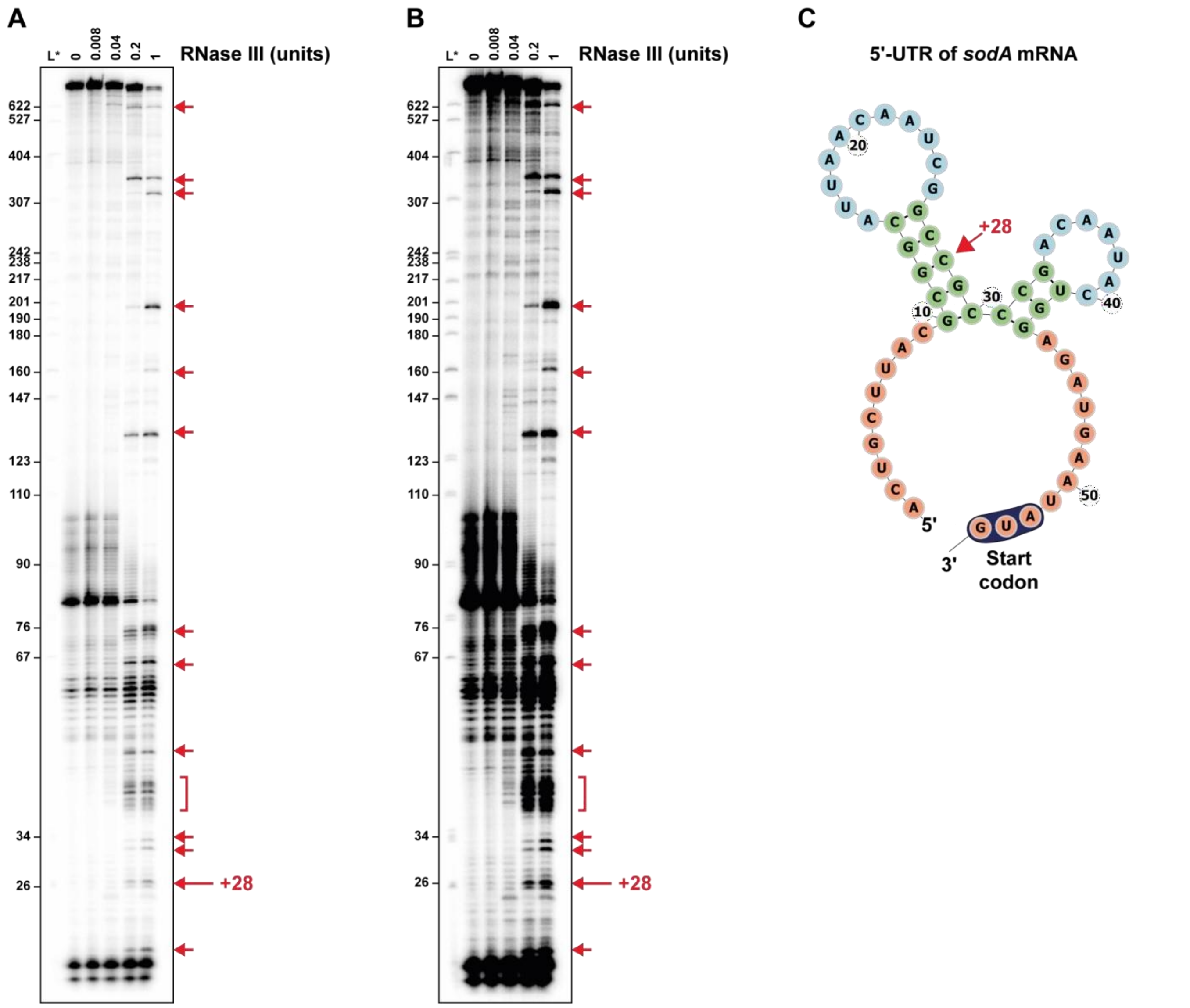

Figure S8. *In vitro* cleavage of *sodA* mRNA by RNase III. A) and B) RNase III digestion of the full-length *sodA* mRNA 5′-radiolabeled at its +1 was performed at 37°C in TMN buffer for 25 min at 37◦C (20 mM Tris acetate, pH 7.5, 10 mM magnesium acetate, 100 mM sodium acetate) containing 1µg yeast tRNA with increasing concentrations of RNase III per sample. Samples were analyzed on 6% polyacrylamide-urea gels. Red arrows indicate the cleavages made by RNase III *in vitro*. Since the full-length *sodA* mRNA was labeled at its 5′-end, the positions of the cleavage sites can be directly inferred from the length of the 5′-radiolabeled marker. The marker (lane L*) is pBR322 digested with MspI (Biolabs). Two exposures of the same gel are shown with (**A**) short time exposure and (**B**) long time exposure. (**C**) The RNase III cleavage site identified at the 28th nucleotide from the transcription start site of *sodA* mRNA, both *in vitro* (this study) and *in vivo* (previous study [84]), is indicated (+28) on the predicted secondary structure of the 55 first nucleotides of the *sodA* mRNA using the RNAfold webserver (The Vienna RNA Websuite, [85]). The start codon (AUG) is highlighted in dark blue. Of note, cleavages observed *in vitro* within the ORF were not observed *in vivo* in a previous study [83], which may be due to additional processing events in the 5′UTR and/or binding of ribosomes preventing RNase III cleavages with in the coding sequence of the *sodA* mRNA.

References

1. Goldblum, K.; Apirion, D. Inactivation of the ribonucleic acid-processing enzyme ribonuclease E blocks cell division. *J. Bacteriol.* **1981**, *146*, 128–132.
2. Regnier, P.; Hajnsdorf, E. Decay of mRNA encoding ribosomal protein S15 of Escherichia coli is initiated by an RNase E-dependent endonucleolytic cleavage that removes the 3' stabilizing stem and loop structure. *J. Mol. Biol.* **1991**, *217*, 283–292.
3. Reuven, N.B.; Deutscher, M.P. Multiple exoribonucleases are required for the 3' processing of Escherichia coli tRNA precursors in vivo. *Faseb J.* **1993**, *7*, 143–148.
4. Korea, C.G.; Badouraly, R.; Prevost, M.C.; Ghigo, J.M.; Beloin, C. Escherichia coli K-12 possesses multiple cryptic but functional chaperone-usher fimbriae with distinct surface specificities. *Environ. Microbiol.* **2010**, *12*, 1957–1977.

5. Sim, S.-H.; Yeom, J.-H.; Shin, C.; Song, W.-S.; Shin, E.; Kim, H.-M.; Cha, C.-J.; Han, S.H.; Ha, N.-C.; Kim, S.W.; et al. *Escherichia coli* ribonuclease III activity is downregulated by osmotic stress: consequences for the degradation of *bdm* mRNA in biofilm formation. *Mol. Microbiol.* **2010**, *75*, 413–425.
6. Korshunov, S.; Imlay, J.A. Two sources of endogenous hydrogen peroxide in *Escherichia coli*. *Mol. Microbiol.* **2010**, *75*, 1389–1401.
7. Altuvia, Y.; Bar, A.; Reiss, N.; Karavani, E.; Argaman, L.; Margalit, H. *In vivo* cleavage rules and target repertoire of RNase III in *Escherichia coli*. *Nucleic Acids Res.* **2018**, *46*, 10380–10394.
8. Lorenz, R.; Bernhart, S.H.; Höner zu Siederdissen, C.; Tafer, H.; Flamm, C.; Stadler, P.F.; Hofacker, I.L. ViennaRNA Package 2.0. *Algorithms Mol. Biol.* **2011**, *6*, 26.
